# Adverse Effects of Hydroalcoholic Extracts and the Major Components in the Stems of* Impatiens balsamina* L. on* Caenorhabditis elegans*

**DOI:** 10.1155/2017/4245830

**Published:** 2017-02-23

**Authors:** Hong-Fang Jiang, Zi-Heng Zhuang, Bei-Wei Hou, Bao-Jun Shi, Cheng-Jie Shu, Lei Chen, Guo-Xin Shi, Wei-Ming Zhang

**Affiliations:** ^1^College of Life Science, Nanjing Normal University, Nanjing 210046, China; ^2^Nanjing Institute for Comprehensive Utilization of Wild Plant, Nanjing 210042, China; ^3^School of Pharmaceutical Engineering and Life Sciences, Changzhou University, Changzhou 213264, China

## Abstract

*Impatiens balsamina* L. (Balsaminaceae), an annual herb found throughout China, has been extensively used in traditional Chinese medicine (TCM). However, our knowledge regarding the adverse effects of* I. balsamina* in vivo is very limited. In this present study, the nematode* Caenorhabditis elegans* model was employed to fully assess the adverse effects of hydroalcoholic (EtOH 55%) extracts of* I. balsamina* stems (HAEIBS) in vivo. After exposure to 10 mg/mL HAEIBS, the major organism-level endpoints of* C*.* elegans* of percent survival, frequency of head thrash and body bends, and reproduction had decreased by 24%, 30%, and 25%, respectively. The lifespan of* C. elegans* was also greatly reduced after HAEIBS exposure compared to the controls. The active compounds in HAEIBS were separated using high speed countercurrent chromatograph (HSCCC) and characterized by high performance liquid chromatography (HPLC) and nuclear magnetic resonance (NMR). Two compounds, lawsone and 2-methoxy-1,4-naphthoquinone (MNQ), and their adverse effects were then more thoroughly detailed in this study. It was found that lawsone is the major toxin in HAEIBS with a higher toxicity than MNQ in terms of negative impact on* C. elegans* mortality, locomotion, reproduction, and lifespan. Our data also suggests that the* C. elegans* model may be useful for assessing the possible toxicity of other Chinese medicines, plant extracts, and/or compounds.

## 1. Introduction

*Impatiens balsamina* L. (Balsaminaceae) is an annual herb found throughout China. Water extracts of* I. balsamina* stems have long been used in Chinese traditional medicine (TCM) for treatment of a wide range of diseases and ailments, including rheumatism, isthmus, generalized body aches, fractures, and fingernail inflammation [1]. A number of compounds have been isolated from the stems of* I. balsamina* and identified, for example, flavonols [2], naphthoquinones [3, 4], anthocyanin [5], and several phenolic compounds [6], and have been shown to have beneficial properties, for example, antioxidant [7], antimicrobial [8], antitumor [9, 10], antianaphylactic [11], anti-inflammatory [12], antipruritic [13], and antinociceptive [14].

Although the use of* I. balsamina* stems has many medical benefits, it may also cause some adverse reactions. For example, long-term exposure to* I. balsamina* stems extracts and compounds has been found to be mildly toxic to several cancer cell lines [15, 16]. Due to this potential toxicity, it is prohibited to prescribe “Tougucao,” a well-known TCM, which is dried aerial stems of* I. balsamina,* to pregnant women [1].

*Caenorhabditis elegans* is a well-known, nonmammalian, alternative animal model that is extensively used in biomedical and toxicological research [17, 18], including assessments of toxicity. Several studies have tested for beneficial and/or adverse effects of compounds isolated from certain plants and Chinese medicines in* C. elegans* [19–23]. This is primarily due to the several advantages of using* C. elegans*, including their short life cycle, ease of handling, small body size, and high sensitivity to toxins and toxicants [17, 24–26]. In addition, the genome is highly conserved between* C. elegans* and mammals, including humans. This level of similarity implies that any assessment of toxicity performed in* C. elegans* might not only be comparable to assessments in other mammalian models, but also applicable to clinical populations [17, 18, 27]. For example, a series of studies found that the toxicity of toxins in* C. elegans* was similar to that observed in mammals [17, 28], implying that the results of toxicological studies performed in* C. elegans* will closely reflect the effects in mammalian models for the majority of compounds tested.

Currently, little is known about the in vivo toxicity and chemical composition of any toxic substances in hydroalcoholic extracts of* I. balsamina* stems (HAEIBS). In this present study, the toxicity of HAEIBS was evaluated in* C. elegans* based on a series of endpoints. To identify the potential compound(s) causing HAEIBS toxicity, we isolated compounds from* I. balsamina* stems using high speed countercurrent chromatography (HSCCC) and then assessed the toxicity of these individual compounds in* C. elegans*. The results of this study will increase our understanding of the in vivo toxicity of* I. balsamina* stems.

## 2. Materials and Methods

### 2.1. Plant Samples and Chemicals

*Impatiens balsamina* L. (Balsaminaceae) plants were collected in July 2014 and authenticated by Dr. Jiu Zhang (Nanjing Institute for Comprehensive Utilization of Wild Plants). A voucher specimen (number 140628) was deposited at the Herbarium of Nanjing Institute for Comprehensive Utilization of Wild Plants. The stems of* I. balsamina* were collected, freeze-dried, and stored at 4°C. All chemicals used in this study were purchased from Sigma-Aldrich (St. Louis, MO, USA).

### 2.2. Preparation of Impatiens Balsamina L. Hydroalcoholic Extracts

Hydroalcoholic was used in our study for the extraction of* I. balsamina* stems based on preliminary experiments demonstrating extractions with 55% ethanol yielded a higher concentration of bioactive substances than extractions with water.

To make HAEIBS, freeze-dried* I. balsamina* stems (50 g) were ground up and passed through a 60-mesh screen to obtain a fine powder and then extracted twice with 400 mL of 55% ethanol with refluxing for 1 hour. The extracting solution was then passed through three layers of filter paper and concentrated by decompressive rotary evaporation at 60°C. The concentrated extracts were completely dried in a freeze-drier and stored at −20°C until later use.

### 2.3. Nematode Maintenance and Assessments of HAEIBS Toxicity

The* C. elegans* wild-type strain N2 was used in this study.* C. elegans* were maintained on plates containing nematode growth medium (NGM) and* Escherichia coli* OP50 at 20°C as previously described [29]. Age synchronous populations of L1 or L4 larvae were obtained using a previously published method [30]. Nematodes at different life stages were exposed to HAEIBS for 48 hours in 12-well plates containing sterile K medium (50 mM NaCl, 30 mM KCl, and 10 mM NaOAc, pH 5.5) and* E. coli* OP50 at 20°C [30, 31]. Nematodes exposed to HAEIBS were then assessed for indicators of toxicity, including lethality, decreases in locomotion, and reduced reproduction (brood size).

The HAEIBS stock solution (500 mg/mL) was prepared in DMSO and then diluted in K medium to a final concentration of 0.1–10 mg/mL before plating. For lawsone and MNQ, the final concentrations were 0.1–0.5 mg/mL.* E. coli* OP50 was spread on the NGM plates as food for the nematodes.

Lethality was evaluated as the percent survival of the population based on a previously described method [32]. Fifty L1 larvae stage* C. elegans* were treated with different concentrations of HAEIBS for 48 hours. Nematodes were considered dead if, while observing under a dissecting microscopy, they failed to respond to mechanical stimulation from a small metal wire. Three replicates were performed for each cohort.

Locomotion was assessed based on both head thrashing and body bending [30, 31]. To assay head thrash, the control and HAEIBS-treated nematodes were washed with K medium and then transferred to a microtiter well containing 60 *μ*L of K medium on top of the agar. After a 1 min recovery period, the number of head thrashes was counted for 1 min, where a thrash was defined as a change in the direction of bending at the mid body. To assay body bend, the nematodes were then placed onto a second plate and the number of body bends was counted for 20 seconds, where a body bend was defined as a change in the direction in the area corresponding to the posterior bulb of the pharynx along the *y*-axis, assuming that the nematode was traveling along the *x*-axis. Three replicates of 15 nematodes were examined for each treatment group.

Reproduction was assessed based on brood size, which was determined as the number of offspring at all stages beyond the egg stage. Nematodes were transferred daily to new agar plates until the end of the egg laying period. Hatched progeny were allowed to grow to the L1/L2 stage and then counted manually. Three replicates of 10 nematodes each were examined for each treatment group.

The lifespan assay was performed as previously described [33, 34]. Briefly, after treating the nematodes for 48 hours starting at the L1 larvae stage, the nematodes were transferred daily to new plates for the first 4 days of adulthood. Nematode viability was checked daily and the nematodes were considered dead when they failed to move after repeated mechanical stimulation with a small metal wire. Fifty nematodes were examined for each treatment group. All graphs display an average of three independent trials.

### 2.4. HAEIBS Chemical Component Isolation and Identification

HAEIBS (15 g) was diluted in 2 L water and then extracted five times with water-saturated light petroleum (b.p. 60–90°C) and ethyl acetate in succession. After analysis by thin layer chromatography (TLC) and high performance liquid chromatography (HPLC), the ethyl acetate fraction was incubated under a vacuum until completely dry and stored at −4°C for subsequent isolation and separation by HSCCC (TBE-300A, Tauto Biotech., Shanghai, China) (*n*-hexane : ethyl acetate : methanol : water = 5 : 4 : 4.5 : 5 (v/v), upper organic phase as the stationary phase, lower aqueous phase as the mobile phase, and flow rate: 1.0 mL/min, 850 rpm, forward). The effluent from the column outlet was continuously monitored at 280 nm. Peak fractions were collected according to the chromatogram and then incubated under a vacuum. A total of 4 peaks, peaks I (4.5 mg), II (10.5 mg), III (13.6 mg), and IV (43.6 mg), were obtained with purities of 96.5, 95.2, 96.8, and 98.3%, respectively, as determined by HPLC-DAD. Each peak fraction was identified using Agilent 1100/MSD electron impact mass spectrometry (EI-MS) (Agilent, California, USA) and a BRUCKER AVANCE 500 nuclear magnetic resonance (NMR) spectrometer (BruckerBioSpin Inc., Switzerland).

### 2.5. Characterization of HAEIBS by HPLC

Crude HAEIBS samples were analyzed with an Agilent 1200 HPLC system (California, US) using a ZORBAX XDB-C_18_ column (150 × 4.6 mm i.d., 5 *μ*m) set at 25°C. The mobile phase was a solution of acetonitrile-2.5% aqueous acetic acid delivered at a flow rate of 1.0 mL/min with a gradient elution of 0–10 min, 25 : 75; 10–20 min, 32 : 68; and 20–35 min, 55 : 45. Samples were injected in a 20 *μ*L volume. The detection wavelength was set at 280 nm.

Separate stock solutions were made in methanol of lawsone, quercetin, MNQ, and kaempferol for the reference standards. A working solution of each standard was prepared in methanol and then diluted to provide a series of standard solutions at concentrations of 50, 40, 25, 12.5, 10, 6.25, and 3.125 *μ*g/mL. The standard curve equations are *Y* = 33.38*X* − 62.247 (lawsone), *Y* = 44.70*X* − 271.25 (quercetin), *Y* = 94.367*X* − 13.345 (MNQ), and *Y* = 56.855*X* + 53.611 (kaempferol).

HAEIBS (10 mg) was dissolved in 10 mL methanol, passed through a 0.45 *μ*m membrane filter, and then analyzed immediately after extraction to avoid possible chemical degradation. All samples were assayed in triplicate.

## 3. Results

### 3.1. HAEIBS Exposure Causes Lethality in* C. elegans*

**Figure 1:**
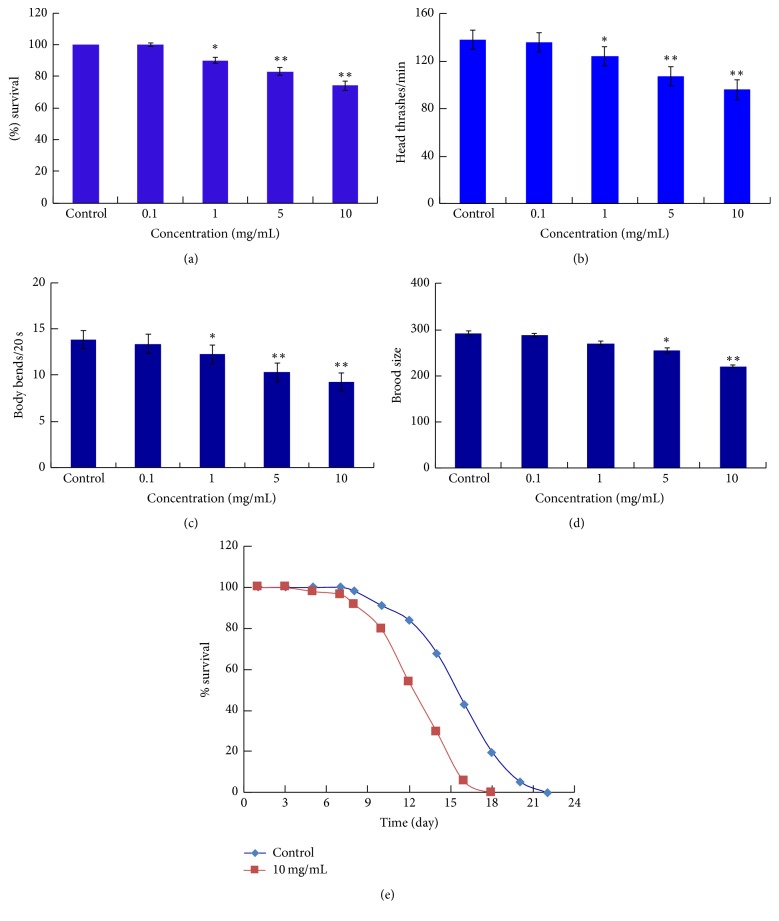
Effects of HAEIBS exposure on* C. elegans*. (a) The survival of L1 larvae after exposure to different concentrations of HAEIBS. *N* = 50. (b) The number of nematodes displaying head thrashing 1 min postexposure to different concentrations of HAEIBS. *N* = 15. (c) The number of nematodes displaying body bending within 20 seconds after HAEIBS treatment. *N* = 15. (d) The number of progeny produced by nematodes treated with different concentrations of HAEIBS. *N* = 10. (e) Effects of 10 mg/mL HAEIBS on nematode lifespan. *N* = 50.* C. elegans* were exposed to HAEIBS for 48 hours spanning the L1 larvae to young adult stages. Bars represent means ± SEM; ^*∗*^*p* < 0.05; ^*∗∗*^*p* < 0.01.

Lethality is a commonly used endpoint when evaluating toxicity in nematodes. To test if HAEIBS exposure is lethal to* C. elegans*, L1 larvae stage nematodes were treated with concentrations of HAEIBS of 0, 0.1, 1, 5, or 10 mg/mL. As shown in Figure 1(a), treatment with 0.1 mg/mL HAEIBS caused no significant lethality in* C. elegans*. However, exposure to 1 mg/mL of HAEIBS moderately decreased nematode survival compared to the controls, while 5 and 10 mg/mL of HAEIBS significantly reduced survival (*p* < 0.01) by 17% and 24%, respectively.

### 3.2. HAEIBS Inhibits* C. elegans* Locomotion

Nematode locomotion is neuronally controlled and includes movements, such as head thrashes and body bends that can be secondary targets of toxins. Therefore, alterations in locomotion are useful for evaluating toxicity in nematodes. It was found that exposure to a low dose of 0.1 mg/mL HAEIBS had no effect on locomotion, but higher doses of 5–10 mg/mL caused a significant decrease in both head thrashes and body bends. Specifically, there was an approximately 30% reduction in head thrashes and body bends after being treated with 10 mg/mL HAEIBS (Figures 1(b) and 1(c)). This data further validates the sensitivity of locomotion to toxins when assessing toxicity in nematodes.

### 3.3. High Doses of HAEIBS Negatively Affect the Reproduction of* C. elegans*

*I. balsamina* stems may contain toxin(s) that negatively impact reproduction as folk medicine has long prohibited the use of “Tougucao,” a herbal medicine containing* I. balsamina*, during pregnancy. Thus, the effect of HAEIBS on the reproduction of* C. elegans* was analyzed using brood size as the endpoint. When the nematodes were treated with 0.1–1 mg/mL of HAEIBS for 48 hours, there was no notable change in their brood size. However, when the concentration of HAEIBS was increased to 5 or 10 mg/mL, the brood size of treated nematodes decreased. Strikingly, exposure to 10 mg/mL HAEIBS led to an approximately 25% reduction in the brood size of treated nematodes (Figure 1(d)).

### 3.4. Exposure to High Concentrations of HAEIBS Reduces the Lifespan of* C. elegans*

Next, the effect of exposure to HAEIBS on the lifespan of* C. elegans* was investigated. Lifespan is an important endpoint as it is likely reflective of any long-term effects of a specific toxin in nematodes. In this study, exposure to 10 mg/mL of HAEIBS significantly decreased the lifespan of nematodes. Specifically, the rate of survival of treated nematodes started to drop 5 days posttreatment, while almost all of the nematodes in control group were still surviving at this time. In addition, the difference in lifespan between the HAEIBS-treated and control nematodes was directly correlated with the number of days posttreatment. By the 10th day, more than 20% of the treated* C. elegans* had died, while more than 90% of the control nematodes were still alive. At 14 days posttreatment, the survival rate of the control group was more than double that of HAEIBS-treated nematodes. Finally, all the HAEIBS-treated* C. elegans* had died by 18 days posttreatment, while about 20% of the control nematodes were still alive. Therefore, this data indicates that exposure to HAEIBS significantly reduces the lifespan of* C. elegans* (Figure 1(e)).

### 3.5. Identification of the Chemical Components in HAEIBS

To determine which compound(s) are causing HAEIBS toxicity, the chemical components in HAEIBS were separated and tested for toxicity. First, HAEIBS components were split into acetyl acetate and light petroleum fractions according to their polarity. After assessing for toxicity, it was noted that only the acetyl acetate fraction had adverse effects on the survival, locomotion, and reproduction of* C. elegans* (unpublished data). This fraction was then further separated by HSCCC. Four major peak fractions were collected from HSCCC and further analyzed by EI-MS, as well as ^1^H-NMR and ^13^C-NMR spectra with TMS-based chemical shifts.

  HSCCC peak I: yellow needle crystal, C_10_H_6_O_3_, m.p. 192–195°C, EI-MS, *m*/*z*: 174 (M^+^), ^1^H-NMR (DMSO-*d*_6_, 500 MHz): *δ*_H_ (ppm): 8.00 (1H, dd, *J* = 7.5 and 1.0 Hz), 7.94 (1H, dd, *J* = 7.5 and 1.0 Hz), 7.84 (1H, td, *J* = 7.45 and 1.2 Hz), 7.80 (1H, td, *J* = 7.45 and 1.2 Hz), 6.19 (1H, s, H-3), and 11.67 (br.s, 2-OH). ^13^C-NMR (DMSO-*d*_6_, 500 MHz): *δ*_C_ (ppm): 184.51 (C-4), 181.10 (C-1), 159.40 (C-2), 134.30 (C-6), 133.08 (C-7), 131.80 (C-10), 130.50 (C-9), 125.80 (C-5), 125.30 (C-8), 111.06 (C-3), which was in accordance with previous reports [6] on lawsone.
  HSCCC peak II: yellow needle crystal, C_15_H_10_O_7_, m.p. 314–317°C, EI-MS, *m*/*z*: 302 (M^+^), ^1^H-NMR (DMSO-*d*_6_, 500 MHz): *δ*_H_ (ppm): 7.72 (1H, s, H-2′), 6.92 (1H, d, *J* = 8.5 Hz, H-5′), 7.58 (1H, d, *J* = 8.5 Hz, H-6′), 6.44 (1H, s, H-8), 6.22 (1H, s, H-6), 12.51 (1H, s, 5-OH), 9.41 (4H, br.s, 3-OH, 7-OH, 3′-OH, 4′-OH). ^13^C-NMR (DMSO-*d*_6_, 500 MHz): *δ*_C_ (ppm): 175.80 (C-4), 163.90 (C-7), 160.70 (C-5), 156.10 (C-9), 146.80 (C-2), 145.01 (C-3′), 115.63 (C-5′), 147.71 (C-4′), 135.70 (C-3), 120.00 (C-1′), 115.10 (C-2′), 122.00 (C-6′), 103.00 (C-10), 98.20 (C-6), 93.37 (C-8), which was in accordance with previous reports on quercetin [6].
  HSCCC peak III: yellow needle crystal, C_11_H_3_O_3_, m.p. 183.5–186°C, EI-MS, *m*/*z*: 188 (M^+^), ^1^H-NMR (DMSO-*d*_6_, 500 MHz): *δ*_H_ (ppm): 8.12 (1H, dd, *J* = 7.4 and 1.5 Hz), 8.08 (1H, dd, *J* = 7.2 and 1.5 Hz), 7.75 (1H, td, *J* = 7.4 and 1.5 Hz), 7.71 (1H, td, *J* = 7.4 and 1.5 Hz), 6.18 (1H, s, H-3), and 3.91 (3H, s). ^13^C-NMR (DMSO-*d*_6_, 500 MHz): *δ*_C_ (ppm): 184.70 (C-4), 180.00 (C-1), 160.40 (C-2), 134.20 (C-6), 133.20 (C-7), 132.00 (C-10), 131.00 (C-9), 126.60 (C-5), 126.10 (C-8), 109.80 (C-3), and 56.3 (C-11), which was in accordance with previous reports on 2-methoxy-1,4-naphthoquinone (MNQ) [4].
  HSCCC peak IV: yellow needle crystal, C_15_H_10_O_6_, m.p. 276–278°C, EI-MS, *m*/*z*: 286 (M^+^), ^1^H-NMR (DMSO-*d*_6_, 500 MHz): *δ*_H_ (ppm): 6.45 (1H, d, *J* = 1.65 Hz, H-8), 6.21 (1H, d, *J* = 1.65 Hz, H-6), 8.06 (2H, d, *J* = 8.8 Hz, H-2′, 6′), 6.94 (2H, d, *J* = 8.8 Hz, H-3′, 5′), 12.49 (1H, s, 5-OH), 10.77 (br.s, 7-OH), 10.09 (br.s, 4′-OH), 9.36 (br.s, 3-OH). ^13^C-NMR (DMSO-*d*_6_, 500 MHz): *δ*_C_ (ppm): 146.80 (C-2), 135.60 (C-3), 175.80 (C-4), 160.70 (C-5), 98.10 (C-6), 163.80 (C-7), 93.40 (C-8), 156.10 (C-9), 103.00 (C-10), 121.60 (C-1′), 129.40 (C-2′, 6′), 115.40 (C-3′, 5′), and 159.10 (C-4′), which was in accordance with previous reports on kaempferol [35].

**Figure 2:**
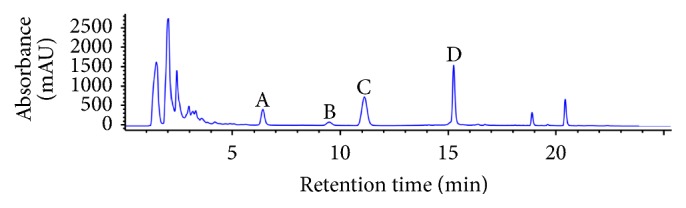
Characterization of HAEIBS by RP-HPLC (*λ* = 280 nm). A: lawsone, B: quercetin, C: MNQ, and D: kaempferol.

Using reverse-phase high performance liquid chromatography (RP-HPLC), the abundances of lawsone, quercetin, MNQ, and kaempferol were measured in HAEIBS (Figure 2) and were 19.32, 2.87, 31.15, and 64.98 mg/g, respectively (Table 1).

### 3.6. Lawsone and MNQ Are the Primary Toxins in HAEIBS

Of the 4 compounds isolated from HAEIBS, quercetin and kaempferol on nematodes have also been examined by our group. These compounds can extend the lifespan and enhance locomotion of* C. elegans* at a concentration of 60 *μ*g/mL. These results are consistent with previous reports in* C. elegans* [36–39] and, thus, are unlikely to mediate the HAEIBS toxicity observed in this study. Therefore, lawsone and MNQ were pursued as the potential cause(s) of HAEIBS toxicity and the toxicity of different concentrations of these two pure compounds was analyzed in* C. elegans*.

**Figure 3:**
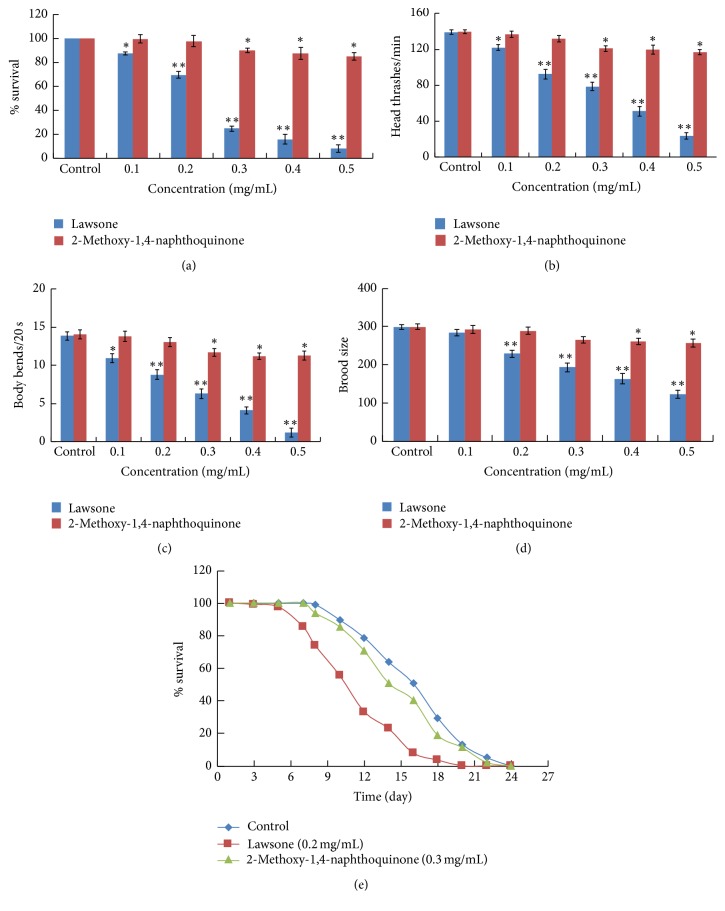
Effects of exposure of lawsone and MNQ on* C. elegans*. (a) The survival L1 larvae after exposure to different concentrations of lawsone or MNQ. *N* = 50. (b) The number of nematodes displaying head thrashing within 1 minute after exposure to different concentrations of lawsone or MNQ. *N* = 15. (c) The number of nematodes displaying body bending within 20 seconds after lawsone or MNQ treatment. *N* = 15. (d) The number of progeny produced by nematodes treated with different concentrations of lawsone or MNQ. *N* = 10. (e) Effects of lawsone and MNQ nematode lifespan. *N* = 50.* C. elegans* were exposed to lawsone or MNQ for 48 hours spanning the L1 larvae to young adult stages. Bars represent means ± SEM; ^*∗*^*p* < 0.05; ^*∗∗*^*p* < 0.01.

As shown in Figure 3(a), exposure to 0.1 mg/mL of lawsone only moderately decreased the survival of nematode compared to the control. However, there was about a 30% decrease in survival for nematodes treated with 0.2 mg/mL lawsone. When treated with 0.5 mg/mL of lawsone, more than 90% of nematodes died, indicating that lawsone is very toxic to* C. elegans*. Although there was no significant lethality observed when nematodes were treated with 0.1 or 0.2 mg/mL of MNQ, exposure to 0.3–0.5 mg/mL of MNQ caused a moderate decrease in nematode survival compared to the controls.

Exposure to a low dose of 0.1 mg/mL of lawsone moderately affected nematode locomotion, while higher doses of 0.2–0.5 mg/mL caused significant decreases in both nematode head thrashing and body bending. There was about a 33% reduction in the amount of head thrashes and body bends after being treated with 0.2 mg/mL lawsone (Figures 3(b) and 3(c)). Exposure to a low dose (0.1–0.2 mg/mL) of MNQ did not affect nematode locomotion, but higher doses (0.3–0.5 mg/mL) led to slight decreases in both nematode head thrashing and body bending (Figures 3(b) and 3(c)).

Lawsone also displayed reproductive toxicity in* C. elegans*. Although a low dose of 0.1 mg/mL had no significant effect on nematode brood size, concentrations of 0.2 mg/mL or higher of lawsone lead to significant decreases in brood size compared to untreated nematodes. Specifically, treatment with 0.2 and 0.5 mg/mL lawsone caused 24% and 50% reductions in brood size, respectively (Figure 3(d)).

Although MNQ did not notably reduce the nematode lifespan at a 0.3 mg/mL concentration, the lifespan of nematode was greatly reduced after treatment with 0.2 mg/mL lawsone. The survival of lawsone-treated nematodes started to drop 6 days after treatment and about 30% of lawsone-treated nematodes died by 8 days posttreatment. At 10 days posttreatment, more than half of the treated* C. elegans* had died compared to only 10% of the control nematodes (Figure 3(e)). By 18 days posttreatment, all lawsone-treated* C. elegans* had died compared to the control nematodes, where 30% were still alive. Therefore, our data indicates that lawsone significantly reduces the lifespan of* C. elegans* and is more toxic than MNQ.

## 4. Discussion

*C. elegans* are invertebrates well suited for use as an animal model due to their short lifecycle, fast reproduction, and well-characterized genome [17]. This model has been successfully used in the assessment of toxicity of heavy metals [40–44], environmental pollutants [45, 46], and specific components of plant extracts [21, 23]. As a whole, these previous studies have resulted in the acceptance of the use of* C. elegans* as a bioindicator in toxicity studies [23, 45, 47, 48]. As further validation, toxicological studies conducted in* C. elegans* have yielded similar results to parallel studies in mammalian models [28]. In our study,* C. elegans* was used to evaluate toxicity of plant extracts and compounds isolated from HAEIBS. Our data further supports the use of* C. elegans* as a rapid and systemic assay system by which to assess toxicity of specific Chinese medicines or plant extracts.

*I. balsamina* stems have long been used in TCM for the treatment of a range of diseases and ailments [7, 13, 14, 49–57]. However, there have been very few studies regarding its potential toxicity [16, 58–63]. Therefore, this present study was designed to comprehensively examine* I. balsamina* stems toxicity and identify any potential toxins. In this work, it was demonstrated that exposure to HAEIBS affected the survival, locomotion, reproduction, and lifespan of* C. elegans*, indicating that* I. balsamina* stems do display toxicity in animals.

In addition, 4 major compounds were isolated from HAEIBS and identified using HSCCC and reverse-phase HPLC, and lawsone and MNQ were found to be the major mediators of HAEIBS toxicity in* C. elegans*. Our data indicates that lawsone at a concentration as low as 0.2 mg/mL displayed significant toxicity in* C. elegans* based on reductions of 30.5% reduction in survival rate, 33.63% in head thrashes per minute, 36.46% in body bends per 20 seconds, and 24% of brood size (Figure 3). Interestingly, similar adverse effects were also observed when* C. elegans* were treated with 10 mg/mL of HAEIBS (Figure 1). Based on the abundance of these compounds in HAEIBS, it was calculated that 1 gram of HAEIBS contains 19.32 mg of lawsone. Therefore, 0.2 mg of pure lawsone is similar to the amount found in 10 mg HAEIBS, clearly suggesting that lawsone is the major toxin in* I. balsamina*. Compared to lawsone, MNQ seemed to be much less toxic as it had no notable toxicity, even at a concentration of 0.5 mg/mL, which is more than 30 times higher than the amount contained in HAEIBS.

**Figure 4:**
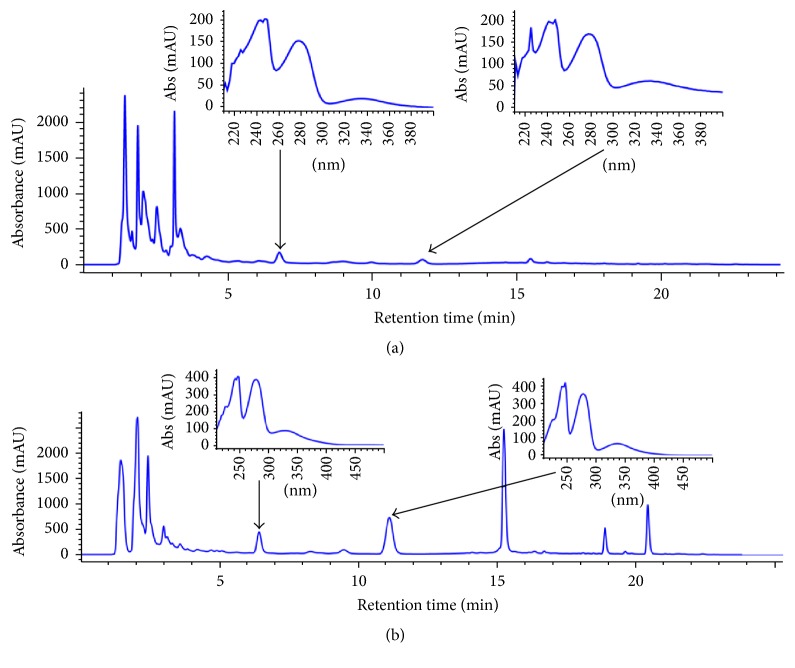
HPLC-DAD of (a) boiling water extracts of* I. balsamina* stems; and (b) hydroalcoholic (EtOH 55%) extracts of* I. balsamina* stems (*λ* = 280 nm). The insets are absorption spectrum of peaks which arrows point to.

The common protocol of preparing most Chinese herb medicine is to decoct herbal material with water for certain time. Then the aqueous part will contain most of the rapeutically active components for treatment. We tried to decoct* I. balsamina* stems powder with boiling water at the beginning. However, due to the poor efficiency of extraction, we could not collect enough samples for further analytical analysis. That was the reason we chose to use hydroalcoholic (EtOH 55%) to do the extraction.  Figure 4 shows the result of HPLC analysis on compounds extracted by either boiling water (Figure 4(a)) or hydroalcoholic (EtOH 55%) (Figure 4(b)) from equal amount of* I. balsamina* stems powder. Clearly, there are huge differences in terms of the abundance of compounds with similar retention time between two different methods. However, peaks with the same retention time from two extractions also have very similar absorption spectrum, suggesting that they are the same compounds. For example, Lawson, the peak at 6.4 minutes in hydroalcoholic (EtOH 55%) extracts, has a corresponding peak with similar absorption spectrum in boiling water extracts, although with much lower signal. Same is true for MNQ. This indicates that the potential toxicants we found in HAEIBS can also be extracted by boiling water. We measured concentration of those four compounds identified from HAEIBS in boiling water extracts of* I. balsamina* stems. Their concentrations are 3.17, 1.73, 0.85, and 1.17 mg/g, respectively. Overall, hydroalcoholic (EtOH 55%) extraction enabled better yield of compounds comparing to boiling water but with much less efficiency. We also measured abundance of lawsone and MNQ in daily dose of “Tougucao” (9–15 g, boiled in water), and 32.4–54 mg of lawsone were detected (unpublished data). Considering 0.2 mg/mL of lawsone had caused toxicity in* C. elegans* and the extreme sensitivity of the human embryo, prohibiting prescribing pregnant women with “Tougucao” is a sound decision.

Likely due to differences in abundance or solubility in the HSCCC solvents, only 6 compounds were isolated from the acetyl acetate fraction of HAEIBS. Four of these were identified successfully (Figure 2). However, the lack of purity and low yield prevented a determination of their structures. Because these 2 compounds did not have any adverse effects on nematodes (unpublished data), we did not study them further. Comparison of the levels of toxicity of raw HAEIBS and pure lawsone and MNQ in nematodes clearly demonstrated that lawsone and MNQ are the primary toxins in HAEIBS. Therefore, even if there are other compounds that were not successfully isolated or identified from HAEIBS via HSCCC, they are unlikely to be major HAEIBS toxins.

## 5. Conclusions

This research studied the potential toxic effects of HAEIBS in vivo and identified lawsone and MNQ as toxins within HAEIBS. Furthermore, it was found that lawsone is the primary toxin, as it is more severely toxic than MNQ. In the future, certain strategies, such as modifying the processing procedure, should be considered to reduce the possible adverse effects of* I. balsamina* stems.

## Figures and Tables

**Table 1:** Characterization of HAEIBS by RP-HPLC.

Compound	Linear range (*μ*g/mL)	*t*_*R*_ (min)	Equation^a^	Linearity (*R*^2^)	Content (mg/g, mean ± SD)
Lawsone	3.25–52.00	6.3~6.7	*Y* = 33.38*X* − 62.247	0.9995	19.32 ± 1.03
Quercetin	7.00–56.00	9.3~9.7	*Y* = 44.70*X* − 271.25	0.9994	2.87 ± 0.82
MNQ	3.625–58.00	11.1~11.6	*Y* = 94.367*X* − 13.345	0.9996	31.15 ± 1.26
Kaempferol	3.625–58.00	15.2~15.7	*Y* = 56.855*X* + 53.611	0.9995	64.98 ± 1.18

^a^ *y* = *ax* + *b*, where *y* is the area of the peak and *x* is the concentration of the analyzed material. *n* = 3 replicates per day with a total *n* = 9 replicates over three separate days for each concentration.
